# The extended clinical and genetic spectrum of *CTNNB1*-related neurodevelopmental disorder

**DOI:** 10.3389/fped.2022.960450

**Published:** 2022-07-22

**Authors:** Seungbok Lee, Se Song Jang, Soojin Park, Jihoon G. Yoon, Soo Yeon Kim, Byung Chan Lim, Jong Hee Chae

**Affiliations:** ^1^Department of Genomic Medicine, Seoul National University Hospital, Seoul, South Korea; ^2^Department of Pediatrics, Seoul National University College of Medicine, Seoul National University Children’s Hospital, Seoul, South Korea

**Keywords:** *CTNNB1*, β-catenin, neurodevelopmental disorder with spastic diplegia and visual defects, exudative vitreoretinopathy 7, Rett-like phenotype, autism spectrum disorder

## Abstract

**Purpose:**

Loss-of-function mutations of *CTNNB1* have been established as the cause of neurodevelopmental disorder with spastic diplegia and visual defects. Although most patients share key phenotypes such as global developmental delay and intellectual disability, patients with *CTNNB1*-related neurodevelopmental disorder show a broad spectrum of clinical features.

**Methods:**

We enrolled 13 Korean patients with *CTNNB1*-related neurodevelopmental disorder who visited Seoul National University Children’s Hospital (5 female and 8 male patients with ages ranging from 4 to 22 years). They were all genetically confirmed as having pathogenic loss-of-function variants in *CTNNB1* using trio or singleton whole exome sequencing. Variants called from singleton analyses were confirmed to be *de novo* through parental Sanger sequencing.

**Results:**

We identified 11 *de novo* truncating variants in *CTNNB1* in 13 patients, and two pathogenic variants, c.1867C > T (p.Gln623Ter) and c.1420C > T (p.Arg474Ter), found in two unrelated patients, respectively. Five of them were novel pathogenic variants not listed in the ClinVar database. While all patients showed varying degrees of intellectual disability, impaired motor performance, and ophthalmologic problems, none of them had structural brain abnormalities or seizure. In addition, there were three female patients who showed autistic features, such as hand stereotypy, bruxism, and abnormal breathing. A literature review revealed a female predominance of autistic features in *CTNNB1*-related neurodevelopmental disorder.

**Conclusion:**

This is one of the largest single-center cohorts of *CTNNB1*-related neurodevelopmental disorder. This study investigated variable clinical features of patients and has expanded the clinical and genetic spectrum of the disease.

## Introduction

Since the first discovery of loss-of-function mutations in intellectual disability patients (1), *CTNNB1* has been established as a causative gene of neurodevelopmental disorder with spastic diplegia and visual defects (NEDSDV), which is characterized by global developmental delay, intellectual disability, facial dysmorphism, and microcephaly (MIM# 615075) (2). This is an autosomal dominant disorder, mostly caused by loss-of-function mutations resulting in disruption of normal molecular function. Although several studies had reported additional cases of NEDSDV, their clinical presentations are different from each other (1, 3–11).

**FIGURE 1:**
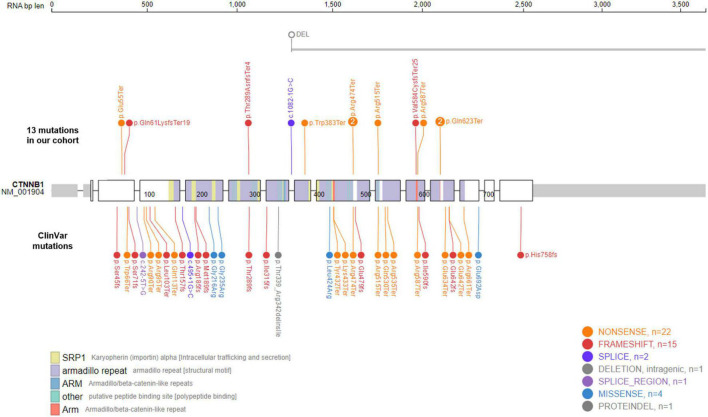
Mutational spectrum of *CTNNB1*-related neurodevelopmental disorder. Eleven *de novo CTNNB1* variants were identified in in 13 patients. Two mutations, c.1867C > T (p.Gln623Ter) and c.1420C > T (p.Arg474Ter), were found in two unrelated patients, respectively. The large deletion of Case 4 spans at least 685 kb including exons 8–15 of *CTNNB1* (NM_001904). We also visualized 33 *de novo* ClinVar mutations in *CTNNB1* together, all of which are predicted to be pathogenic or likely pathogenic.

The *CTNNB1* gene encodes the protein β-catenin, a key component of the Wnt signaling pathway. Its roles in carcinogenesis have been well established in many different types of cancers, including colorectal cancer (MIM# 114500), hepatocellular carcinoma (MIM# 114550), medulloblastoma (MIM# 155255), ovarian cancer (MIM# 167000), and pilomatricoma (MIM# 132600) (2). Cancer-causing mutations in *CTNNB1* are mostly somatic, gain-of-function mutations. Currently, 28 oncogenic or likely oncogenic *CTNNB1* variants are listed in the OncoKB database,^1^ and all of them are missense or gain-of-function mutations (12). In contrast, most of variants causing NEDSDV are *de novo*, loss-of-function mutations. There are 33 *de novo CTNNB1* variants listed in the ClinVar database to date,^2^ all of which are predicted to be pathogenic or likely pathogenic (13). Among these, 27 variants are loss-of-function mutations including 14 nonsense, 12 frameshift, and 1 canonical splicing site variants (Figure 1).

In addition to NEDSDV and several kinds of cancers mentioned above, the defects in *CTNNB1* can also result in the ophthalmologic disorder, exudative vitreoretinopathy 7 (MIM# 617572). Panagiotou et al. reported that Mendelian inherited *CTNNB1* mutations can cause non-syndromic familial exudative vitreoretinopathy (14). They reported one missense mutation (p.Arg710Cys) and one truncating mutation (p.His720Ter) located in the carboxy-terminal domain of the β-catenin protein. Recent studies also associated missense or truncating variants of *CTNNB1* with vitreoretinopathy and suggested that ophthalmologic examination should be performed in every patient with *CTNNB1*-related disorders (15, 16).

Advances in sequencing technology have greatly improved genetic diagnosis in clinical practice. Whole exome sequencing (WES) or targeted gene panel sequencing is generally used, and multiple lines of evidence have already demonstrated its efficacy as a first-tier or second-tier genetic test in various kinds of diseases (17–19). Trio WES is an especially efficient diagnostic strategy for patients with neurodevelopmental disorders but without known etiologies because it enables detection of *de novo* or compound heterozygous variants (20).

In this study, we report clinical presentations of 13 Korean NEDSDV patients, whose *CTNNB1* loss-of-function mutations were identified using singleton or trio WES analyses. This is one of the largest single-center cohorts of NEDSDV, expanding the clinical and genetic spectrum of *CTNNB1*-related neurodevelopmental disorders.

## Materials and methods

### Study participants

We enrolled 13 Korean NEDSDV patients who visited the pediatric neurology clinic of Seoul National University Children’s Hospital. They were genetically confirmed as having *de novo* loss-of-function mutations in *CTNNB1*, which were not found in their parents. The medical records of the patients were retrospectively reviewed by a pediatric neurologist. The study was performed in accordance with the ethical standards of the Declaration of Helsinki and was approved by the Institutional Review Board of Seoul National University Hospital (#2003-192-1112 and #1406-081-588).

### Genetic diagnosis

*CTNNB1* mutations were detected through next generation sequencing, seven patients by trio WES and six patients by singleton WES analyses. Except Cases 1, 2, 12, and 13 who were sequenced in other hospitals or laboratories, we conducted WES using the Illumina technology and the detailed WES methods were described in our previous study (21). WES data were aligned to the reference genome hg19 and processed according to the best practice of Genome Analysis Toolkit (22). We used the ANNOVAR program for variant annotation, such as the RefSeq gene set and gnomAD (23, 24), and focused on rare protein-altering variants (< 0.001% frequency in gnomAD). The Human Gene Mutation Database^3^ and ClinVar databases were screened to check whether identified variants were previously reported (13, 25).

We further selected *de novo* variants in trio WES analyses, and candidate variants called from singleton WES were confirmed to be *de novo* through parental Sanger sequencing. Genomic DNA was extracted from blood using the QIAamp DNA Blood Midi Kit (Qiagen, Hilden, Germany) and PCR was performed using Solg™ 2X h-Taq PCR Smart mix (Solgent, Daejeon, Korea) according to the manufacturers’ instructions. All primers were synthesized by the Bioneer Company (Daejeon, Korea) and PCR products were sequenced by Macrogen (Seoul, Korea).

We used the Moralizer tool^4^ to check the description and predicted protein change for each candidate variant (26), and visualized them using the PeCan protein viewer^5^ (27). Identified variants were described in accordance with the Human Genome Variation Society guidelines (28).

## Results

### *CTNNB1* loss-of-function mutations discovered in our cohort

We identified 11 different *CTNNB1* variants in 13 patients, 8 nonsense, 3 frameshift, 1 canonical splicing site, and 1 large deletion mutation (Table 1 and Figure 1). All of them were confirmed as *de novo* variants by parental analysis and predicted to be pathogenic according to the American College of Medical Genetics and Genomics criteria. They were not reported in gnomAD (allele frequency = 0), and five of them were novel pathogenic variants not listed in ClinVar or HGMD (Supplementary Table 1 and Supplementary Figure 1).

**TABLE 1:** Neurodevelopmental disorder patients with *de novo* loss-of-function mutations in *CTNNB1* (NM_001904.4).

Sample	Gender/Age* (y)	Variant details/ClinVar reported	UMN signs	Last motor status	Last language status (FSIQ)	Behavioral problems	Autistic features	Brain MRI/Seizure	Ophthalmologic features
Case 1	M/5	c.163G>T (p.Glu55Ter)/yes	Yes	Walks with support, spastic gait	<10 single words	No	No	WNL/none	Rt tilted optic disc, anisometropia
Case 2	F/20.5	c.181del (p.Gln61LysfsTer19)/no	Yes	Walks alone, spastic and mildly ataxic gait	Sentences, possible to read (40)	No	No	WNL/none	Lt microphthalmia, PHPV, ptosis, strabismus
Case 3	M/7	c.863_864insG (p.Thr289AsnfsTer4)/no	No	Walks alone, dyspraxic gait	<10 single words	No	No	WNL/none	Strabismus
Case 4	M/11	Exon 8-15 deletion/no	Yes	Walks alone, spastic and tiptoe gait	Sentences, possible to read, dysarthria	Yes (attention deficit, hyperactivity)	No	WNL/none	Strabismus
Case 5	F/8.5	c.1082-1G>C/yes	No	Walks with support	No words	Yes (aggressive behavior, impulsivity)	Yes (hand stereotypy, bruxism, breathing irregularity)	WNL/none	Strabismus
Case 6	M/10	c.1148G>A (p.Trp383Ter)/no	Yes	Walks alone, tiptoe gait	Sentences, possible to read	Yes (aggressive behavior, impulsivity)	No	WNL/none	Both hyperopia, strabismus
Case 7	M/9.5	c.1420C>T (p.Arg474Ter)/yes	Yes	Walks alone, tiptoe, and ataxic gait	Sentences, possible to read (46)	Yes (aggressive behavior)	No	WNL/none	Lt microphthalmia, PHPV, strabismus
Case 8	F/10	c.1759C>T (p.Arg587Ter)/yes	No	Walks alone, tiptoe, and ataxic gait	No words	Yes (aggressive behavior)	Yes (hand stereotypy, bruxism)	WNL/none	Rt ptosis, strabismus
Case 9	M/8	c.1867C>T (p.Gln623Ter)/yes	Yes	Sits with support	No words except mama and papa	No	No	WNL/none	Both optic nerve hypoplasia, Lt myopia
Case 10	M/7.5	c.1867C>T (p.Gln623Ter)/yes	Yes	Walks with support	Sentence, dysarthria	Yes (hyperactivity)	No	WNL/none	Strabismus
Case 11	F/6	c.1749dup (p.Val584CysfsTer25)/no	Yes	Walks alone, tiptoe gait	<10 single words	No	No	WNL/none	Lt PHPV, strabismus
Case 12	F/8.5	c.1420C>T (p.Arg474Ter)/yes	No	Walks alone, tiptoe, and mildly ataxic gait	Sentences, possible to read (58)	No	Yes	WNL/none	NA
Case 13	M/4	c.1543C>T (p.Arg515Ter)/yes	Yes	Sits with support	No words except mama and papa	No	No	WNL/none	Both mild hyperopia

M, male; F, female; WNL, within normal limit; Rt, right; Lt, left; PHPV, persistent hyperplastic primary vitreous; UMN, upper motor neuron; FSIQ, full-scale intelligence quotient. *Age at last follow-up.

The proportion of nonsense variants (61.5%, 8 out of 13) was higher than that of ClinVar (42.4%, 14 out of 33). All identified mutations in our cohort were loss-of-function mutations, and the large deletion of Case 4, spanning at least 685 kb from exon 8 to exon 15, is also predicted to result in an out-of-frame mutation. Like other kinds of mutations causing diseases by loss-of-function mechanisms, *CTNNB1* mutations in our patients were located throughout the gene without any identifiable hot spots. Two mutations, c.1867C > T (p.Gln623Ter) and c.1420C > T (p.Arg474Ter), were found in two unrelated patients, respectively.

### The clinical spectrum of *CTNNB1*-related neurodevelopmental disorder

We have summarized the clinical features of our patients with *CTNNB1* mutations in Table 1. While most patients shared the key phenotypes of NEDSDV, such as developmental delay and intellectual disability, they also showed a broad spectrum of clinical presentations particular to individual patients. All patients in our cohort were 4 years of age or older and diagnosed with neurodevelopmental disorder by pediatric neurologists. While Cases 5 and 8 could not speak any meaningful words until nearly 10 years of age, almost half of the patients could speak sentences and read some words or sentences. Among them, full-scale intelligence quotients were evaluated in three patients, with results ranging from 40 (Case 2) to 58 (Case 12).

Motor development also differed from patient to patient. While Cases 9 and 13 could not sit alone at their last follow-up, others could walk with or without support. Although more than half of the patients (61.5%, 8 out of 13) were able to walk alone without any assistance, all of them showed abnormal gait patterns such as spastic, tiptoe, and ataxic gaits. Similarly, upper motor neuron signs such as increased muscle tone, increased deep tendon reflexes, and Babinski signs were evident in 9 of the 13 patients (69.2%).

Twelve patients were evaluated by ophthalmologists and they all showed various kinds of ocular abnormalities. Strabismus, shown in 9 patients (69.2%), was the most common ophthalmologic problem in our cohort. Unilateral persistent hyperplastic primary vitreous (PHPV) was shown in 3 patients, and two of them also had microphthalmia on the affected side.

**FIGURE 2:**
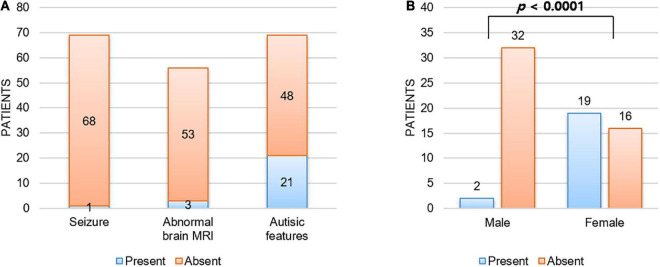
Unique clinical manifestations of *CTNNB1*-related neurodevelopmental disorder. **(A)** Patients with *CTNNB1*-related neurodevelopmental disorder were less likely to have seizure (1.4%) or structural brain abnormality (5.4%), and autistic features were shown in 30.4% of patients. Here we considered three patients with borderline brain MRI findings (delayed bilateral frontal lobe myelination, mild corpus callosum thinning, and mild dilation of ventricles) as normal. In addition, abnormal brain MRI findings noted in two patients out of three also did not suggest obvious structural abnormality (left temporal lobe atrophy and hypoplasia of corpus callosum). **(B)** Autistic features were significantly more frequent in female patients than in male patients. Fisher’s exact test was used to compare frequency of autistic features by gender.

Although every patient in our cohort underwent brain MRI and their images were reviewed by pediatric radiologists, there were no remarkable abnormalities noted in their brains. In addition, none of our patients had any seizure history during follow-up. As shown in Figure 2A, we reviewed a total of 69 patients with *CTNNB1*-related neurodevelopmental disorder reported in the literature, including our patients, and found that only one (1.4%) and three (5.4%) patients had seizure history and brain abnormalities, respectively (1, 3–11, 13–15). The patient with seizure history was suspected as having absence seizure in early childhood. However, her electroencephalogram was normal, and it was uncertain that such events were true clinical seizures. In addition, while one patient had definite brain abnormalities including dysgenesis of the corpus callosum, absence of the right fornix, and hypoplastic brainstem, the brain MRI findings of the other two were just left temporal lobe atrophy and hypoplasia of the corpus callosum, respectively. Therefore, although every patient had apparent developmental delay or intellectual disability, seizure or structural brain abnormality would be an uncommon phenotype of *CTNNB1*-related neurodevelopmental disorder or NEDSDV.

### Autistic features are predominantly found in female patients

In addition to the above clinical presentations, there were 6 patients (46.2%, 4 males and 2 females) who showed behavioral problems; aggressive behavior in 4 patients, hyperactivity in 2 patients, and impulsivity in 2 patients (Table 1). Rett-like or autistic features were also shown in 3 patients, presenting with symptoms such as hand stereotypy, bruxism, and irregular breathing.

Interestingly, all three patients with autistic features were female in this study. Autistic features have been reported frequently (1, 3–11, 13–15), shown in around 30% of patients with *CTNNB1*-related neurodevelopmental disorder (Figure 2A). Consistent with our study, autistic features were significantly more frequent in female patients (54.3%, 19 out of 35) than in male patients (5.9%, 2 out of 34) with a *P*-value = 1.3 × 10^–5^ by Fisher’s exact test (Figure 2B).

Although we searched for other modifying factors determining clinical manifestations of *CTNNB1*-related neurodevelopmental disorder, such as mutation type and location, gender was the only significant factor associated with autistic features in our analysis. In particular, there were two pairs of unrelated patients sharing the same mutation (p.Gln623Ter and p.Arg474Ter), but their clinical presentations were different from each other despite the shared genotype (Table 1).

## Discussion

In this study we summarized the clinical presentations of 13 NEDSDV or *CTNNB1*-related neurodevelopmental disorder patients in our center. Eleven kinds of *de novo* mutations were identified, 5 of them being novel mutations not listed in ClinVar or HGMD. This is one of the largest single-center cohorts of NEDSDV, expanding the clinical and genetic spectrum of the disease.

As already linked with NEDSDV (MIM# 615075) and exudative vitreoretinopathy 7 (MIM# 617572), clinical features such as developmental delay, intellectual disability, spastic diplegia, and ocular problems have been well established in *CTNNB1*-related neurodevelopmental disorder (1, 3–8). Although most of our patients shared such key phenotypes, the severity of each symptom was different patient by patient. While half of the patients could speak sentences and read some words or sentences, two patients (Cases 2 and 12) could not speak any meaningful words at all. While most patients could walk with or without support, two patients (Cases 9 and 13) never achieved sitting or standing without support during follow-up. Likewise, ophthalmologic features also varied from strabismus to PHPV in our patients. Although we searched for associations between mutation type or location and disease phenotype, we could not find any significant genotype-phenotype correlations in our cohort. A recent study also reported that no genotype-phenotype correlations have been identified in *CTNNB1*-related neurodevelopmental disorder to date (15, 29).

In an animal study using a β-catenin conditional knockout mouse, the authors found an alteration in the β-catenin pathway involving cadherin-based synaptic adhesion complexes, which are essential for normal brain function (30). Although there were patients with non-syndromic familial exudative vitreoretinopathy inherited in an autosomal dominant manner, these mutations were missense or truncating mutations located in the carboxy-terminal domain of β-catenin (14). Most patients with loss-of-function mutations in *CTNNB1* showed varying degrees of intellectual disability. In this study, Cases 2, 7, and 12 showed milder intellectual disability compared to the other patients. However, their full-scale intelligence quotient scores were as low as 40, 46, and 58, respectively. This finding suggests that it is quite difficult to expect normal or tolerable intelligence in patients with loss-of-function *CTNNB1* mutations.

The association between seizure and developmental delay or intellectual disability has been well established, and one study reported the prevalence of epilepsy in global developmental delay patients as high as 56% (31–33). Among the 69 patients with *CTNNB1*-related neurodevelopmental disorder we reviewed, none of them had apparent seizure history or abnormal electroencephalogram results (1, 3–11, 13–15). In addition, while structural brain abnormalities have been detected in around 30% of developmental delay patients (34, 35), there were only three patients (5.4%) showing remarkable brain abnormalities among 56 patients. Therefore, we hypothesize that such a low frequency of seizure or brain abnormality might be rather a clinical characteristic of *CTNNB1*-related neurodevelopmental disorder.

Autistic or Rett-like phenotypes have been commonly reported in patients with *CTNNB1*-related neurodevelopmental disorder (1, 3–11, 13–15), and recent studies suggest that the Wnt signaling pathway is one of the major developmental pathways affecting autistic behaviors and could be a therapeutic target of the disease (36, 37). *CTNNB1* is a key regulator of the canonical Wnt pathway and plays a key role in neurodevelopment. Interestingly, by analyzing patients of previous studies including those of our cohort, we revealed that autistic features were predominantly found in female NEDSDV patients. There might be some gender differences in molecular pathways that result in female predominance. Further studies are required to confirm this tendency and reveal its underlying mechanisms. On the other hand, six of our patients and several patients in other studies had various kinds of behavior problems. Such clinical features need to be investigated in more patients with *CTNNB1*-related neurodevelopmental disorder.

Mutations causing disease by a loss-of-function mechanism were generally located throughout the gene rather than concentrated in a hot spot. However, two mutations identified in our cohort, c.1867C > T (p.Gln623Ter) and c.1420C > T (p.Arg474Ter), were found in two unrelated patients, respectively (Figure 1). These amino-acid positions might be fragile sites of *CTNNB1*, and further studies are required to investigate such mutation-prone sequences in the human genome. On the other hand, as shown in Table 1, their clinical features were different from each other despite having identical mutations. We suggest that there are other genetic and/or environmental factors that modify the clinical presentation, such as gender or other genes involved in the Wnt/β-catenin signaling pathway.

This study presented the genetic and clinical spectrum of *CTNNB1*-related neurodevelopmental disorder and identified some key clinical features of the disease. More clinical studies are required to validate our findings, and molecular studies are also needed to discover novel therapeutic targets for treatment of neurodevelopmental disorders.

## Data Availability Statement

The data presented in this study are deposited in the National Center for Biotechenology Information (NCBI) BioProject repostitory, accession number: PRJNA855946.

## Ethics statement

The studies involving human participants were reviewed and approved by Institutional Review Board of Seoul National University Hospital. Written informed consent to participate in this study was provided by the participants’ legal guardian/next of kin.

## Author contributions

BL and JC: study conception and design. SK, BL, and JC: data collection. SL, SJ, JY, and SP: data analysis. SL and JC: results interpretation. SL: drafting the manuscript. All authors contributed to the manuscript revision and approval.

## Conflict of Interest

The authors declare that the research was conducted in the absence of any commercial or financial relationships that could be construed as a potential conflict of interest.

## Publisher’s Note

All claims expressed in this article are solely those of the authors and do not necessarily represent those of their affiliated organizations, or those of the publisher, the editors and the reviewers. Any product that may be evaluated in this article, or claim that may be made by its manufacturer, is not guaranteed or endorsed by the publisher.

## Footnotes

1
https://www.oncokb.org/

2
https://www.ncbi.nlm.nih.gov/clinvar/

3
http://www.hgmd.cf.ac.uk/

4
https://mutalyzer.nl/

5
https://pecan.stjude.cloud/proteinpaint/
