# Supplementary figures and images for: The extended clinical and genetic spectrum of CTNNB1-related neurodevelopmental disorder
Source: Front Pediatr. 2022 Jul 22;10:960450. doi: 10.3389/fped.2022.960450 (PMC9353113; doi:10.3389/fped.2022.960450)

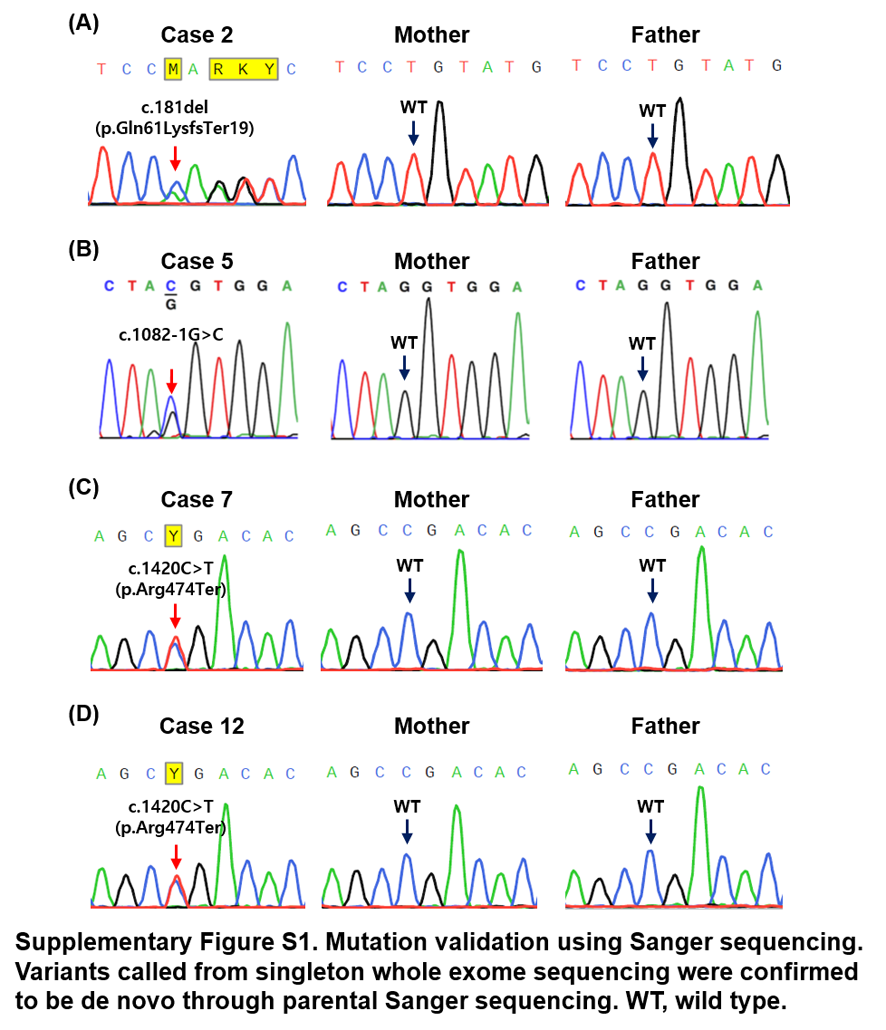

Supplement: Supplementary file 1 [file Image_1.TIF]
